# Pituitary adenoma imaging as a determinant of acromegaly diagnosis and outcomes

**DOI:** 10.1210/clinem/dgag179

**Published:** 2026-04-24

**Authors:** Linus Haberbosch, James MacFarlane, Daniel Gillett, Sophie Howarth, Jonathan Jones, Heok Cheow, Vanessa Hubertus, Güliz Acker, Leontine Bakker, Marco Verstegen, Lenka Pereira Arias-Bouda, Nienke Biermasz, Michael Buchfelder, Christian J Strasburger, Mark Gurnell

**Affiliations:** Cambridge Endocrine Molecular Imaging Group, Metabolic Research Laboratories, Institute of Metabolic Science, University of Cambridge, and National Institute for Health Research Cambridge Biomedical Research Centre, Addenbrooke’s Hospital, Cambridge Biomedical Campus, Cambridge CB2 0QQ, UK; Charité – Universitätsmedizin Berlin, Department of Endocrinology and Metabolism, European Reference Network on Rare Endocrine Conditions (Endo-ERN), Berlin 10117, Germany; BIH Biomedical Innovation Academy, BIH Charité Junior Digital Clinician Scientist Program, Berlin Institute of Health at Charité – Universitätsmedizin Berlin, Berlin 10117, Germany; Cambridge Endocrine Molecular Imaging Group, Metabolic Research Laboratories, Institute of Metabolic Science, University of Cambridge, and National Institute for Health Research Cambridge Biomedical Research Centre, Addenbrooke’s Hospital, Cambridge Biomedical Campus, Cambridge CB2 0QQ, UK; Cambridge Endocrine Molecular Imaging Group, Metabolic Research Laboratories, Institute of Metabolic Science, University of Cambridge, and National Institute for Health Research Cambridge Biomedical Research Centre, Addenbrooke’s Hospital, Cambridge Biomedical Campus, Cambridge CB2 0QQ, UK; Department of Nuclear Medicine, Addenbrooke’s Hospital, Cambridge Biomedical Campus, Cambridge CB2 0QQ, UK; Cambridge Endocrine Molecular Imaging Group, Metabolic Research Laboratories, Institute of Metabolic Science, University of Cambridge, and National Institute for Health Research Cambridge Biomedical Research Centre, Addenbrooke’s Hospital, Cambridge Biomedical Campus, Cambridge CB2 0QQ, UK; Department of Radiology, Addenbrooke’s Hospital, Cambridge Biomedical Campus, Cambridge CB2 0QQ, UK; Department of Nuclear Medicine, Addenbrooke’s Hospital, Cambridge Biomedical Campus, Cambridge CB2 0QQ, UK; Department of Radiology, Addenbrooke’s Hospital, Cambridge Biomedical Campus, Cambridge CB2 0QQ, UK; Department of Neurosurgery, Charité – Universitätsmedizin Berlin, Berlin 10117, Germany; BIH Biomedical Innovation Academy, BIH Charité Clinician Scientist Program, Berlin Institute of Health at Charité – Universitätsmedizin Berlin, Berlin 10117, Germany; Department of Neurosurgery, Charité – Universitätsmedizin Berlin, Berlin 10117, Germany; BIH Biomedical Innovation Academy, BIH Charité Clinician Scientist Program, Berlin Institute of Health at Charité – Universitätsmedizin Berlin, Berlin 10117, Germany; Department of Endocrinology, Leiden University Medical Centre, Leiden 2333 ZA, The Netherlands; Department of Neurosurgery, Leiden University Medical Centre, Leiden 2333 ZA, The Netherlands; Department of Radiology & Nuclear Medicine, Leiden University Medical Centre, Leiden 2333 ZA, The Netherlands; BIH Biomedical Innovation Academy, BIH Charité Clinician Scientist Program, Berlin Institute of Health at Charité – Universitätsmedizin Berlin, Berlin 10117, Germany; Department of Neurosurgery, University of Erlangen-Nürnberg, Erlangen 91054, Germany; Charité – Universitätsmedizin Berlin, Department of Endocrinology and Metabolism, European Reference Network on Rare Endocrine Conditions (Endo-ERN), Berlin 10117, Germany; Cambridge Endocrine Molecular Imaging Group, Metabolic Research Laboratories, Institute of Metabolic Science, University of Cambridge, and National Institute for Health Research Cambridge Biomedical Research Centre, Addenbrooke’s Hospital, Cambridge Biomedical Campus, Cambridge CB2 0QQ, UK

**Keywords:** acromegaly, magnetic resonance imaging, molecular imaging, positron emission tomography

## Abstract

In the majority (>95%) of patients with acromegaly, the underlying cause is a somatotroph adenoma. Pituitary-targeted surgery and radiotherapy can achieve long-term disease control but carry a significant adverse risk to the remaining normal gland, especially if undertaken outside a Pituitary Tumor Center of Excellence or when repeat surgery is performed. Maximizing benefits and minimizing the risks of pituitary surgery and radiotherapy is critically dependent on high-quality imaging that allows accurate localization of site(s) of either de novo, residual, or recurrent disease. Macroadenomas continue to predominate among somatotroph tumors (70%), but more widespread use of intracranial imaging leads to earlier detection of smaller adenomas, posing new challenges for imaging diagnostics.

Despite several comprehensive guidelines on the management of acromegaly, there is little consensus regarding optimal imaging, and current scanning protocols remain heterogeneous. This serves as a barrier to optimal patient management and constrains comparison between centers, with inevitable consequences for research within the field. Here, based on a comprehensive review of previous studies and focusing on recent advances in magnetic resonance and molecular (functional) imaging techniques, we propose a standardized, tiered approach to pituitary imaging in patients with acromegaly, guided by individual patient features and tailored to the anticipated therapeutic approach. An online tool, which can be adapted to the clinical context, is provided as an aid to decision-making.

Acromegaly is a rare but debilitating disease, with an estimated incidence of 3 to 4 cases per million per year and a prevalence of between 40 and 70 cases per million (1). Untreated acromegaly predisposes one to an array of complications, including premature cardiovascular disease, hypertension, diabetes mellitus, sleep apnea, metabolic bone disease, and arthropathy (2). It has also been linked to an increased risk of some cancers (eg, colon) (2). In >95% of patients with acromegaly, the underlying cause is a benign somatotroph pituitary adenoma (1). Somatotroph adenomas are estimated to comprise 13.2% to 16.5% of all pituitary adenomas (3-5).

The reported prevalence of acromegaly has increased over the last decade (6) and likely reflects greater disease awareness, improved diagnostic pathways, and advances in treatment, which have led to increased survival. The delay between symptom onset and diagnosis has also decreased over time, from estimates of >20 years in patients diagnosed before 1990 to approximately 5 years in the 2000s (7), and more recently reported as 2.5 years (8, 9).

Although macroadenomas predominate among somatotroph tumors (∼70%) (6), increased screening of at-risk groups (eg, those harboring mutations in *MEN1* and *AIP*) and more widespread use of intracranial imaging may lead to earlier diagnosis and thereby partially redress the balance between micro- and macro-adenomas. Early detection of smaller lesions should, in turn, improve the prospect of successful treatment and lessen the risk of developing comorbidities because cure rates of the most experienced pituitary surgeons are 80% to 90% for microadenomas and 50% to 60% for macroadenomas (8).

However, while earlier detection of acromegaly would be anticipated to result in better clinical outcomes, smaller somatotroph adenomas potentially pose new challenges for imaging diagnostics (10). In addition, with the advent of advanced endoscopic surgical techniques and increased use of image guidance, larger invasive macroadenomas—including some with extrasellar extension—are now amenable to curative resection (11). In both of these contexts, high-quality imaging of the sella region is critical to accurately locate the sites of active disease and is especially important when repeat surgery or stereotactic radiosurgery are being considered (12, 13).

Advances in magnetic resonance imaging (MRI) (eg, use of higher field strength, adoption of novel sequences), coupled with the advent of pituitary molecular (functional) imaging using positron emission tomography (PET), have enabled more patients to be offered definitive treatment with the anticipation of sparing/reducing the need for long-term medical therapy (14). In parallel, imaging may also provide important insights when selecting which medical therapy to recommend for an individual patient (eg, via prediction of likely response to first- or second-generation somatostatin receptor ligand [SRL] therapy) (15). However, significant variations in imaging protocols, both within (eg, during follow-up of individual patients) and between centers, not only impact routine clinical care but also constrain scientific progress toward defining what constitutes optimal imaging for patients with somatotroph adenomas (16-18). If patients are to gain maximal benefit from earlier diagnosis and improved surgical techniques, it is important that developments in pituitary imaging keep pace and are applied uniformly.

Recent Acromegaly Consensus Group (ACG) guidelines setting out criteria for diagnosis and remission recommend a more harmonized approach to pituitary imaging to address these concerns (19). Building on these recommendations, and based on a comprehensive review of the literature, we now propose a tiered imaging approach that is informed by initial radiologic findings and pays particular attention to the key clinical question(s) under consideration. If widely adopted, such an approach would ensure a minimum clinical standard, facilitate comparison across centers, reduce costs associated with unnecessary repeat imaging, and enable aggregation of results from different centers to address important research questions. To improve accessibility of the algorithm, we have created a website hosted on Bluehost (Bluehost, Jacksonville, Florida) with the algorithm embedded as an online tool using WordPress.com and the Typeform WordPress plugin (Typeform S.L., Barcelona, Spain). Finally, we provide an outlook on future developments in imaging for somatotroph adenomas.

## Literature review

**Figure 1:**
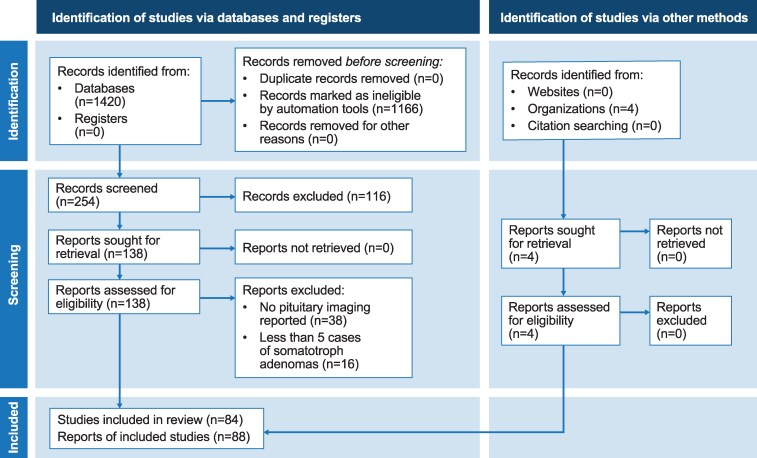
PRISMA chart of the systematic review. Abbreviation: PRISMA, Preferred Reporting Items for Systematic Reviews and Meta-Analyses.

A literature search in MEDLINE via PubMed was performed using the terms (“Acromegaly”[Mesh] OR “Growth Hormone-Secreting Pituitary Adenoma”[Mesh]) AND (“Magnetic Resonance Imaging”[Mesh] OR “Multimodal Imaging”[Mesh] OR “Positron Emission Tomography Computed Tomography”[Mesh] OR “Tomography, X-Ray Computed”[Mesh]). The timeframe considered was October 1, 1971 (reflecting the date of the first computed tomography [CT] scan) to December 31, 2024. The following study types were considered: clinical trial, guideline, meta-analysis, practice guideline, and randomized controlled trial. Papers written in English, German, French, Spanish, or Polish were included. Publications were excluded if they reported no imaging data or included < 5 cases of somatotroph adenomas. Of 1420 studies initially screened, 138 remained after filtering and screening for study type and language, and these were subjected to detailed analyses of reported imaging data; 88 publications met the criteria, of which 57 were included in the final list of MRI studies. The PRISMA chart is provided as Fig. 1, and the complete final list of MRI studies reviewed for the development of this article is reported in Table 1. The 4 relevant consensus guidelines from the American Association of Clinical Endocrinology (2011) (76), the Endocrine Society (2014) (77), the Pituitary Society (2021) (78), and the ACG (2024) (19) were also included.

**Table 1:** Reported MRI protocols for somatotroph adenomas: systematic review

Study	N	Tesla	Sequences	Orientation	Contrast
van der Lely, Biller, Brue, et al (20)	1288	—	T1, T2 FSE	c/s	Yes
Brue, Castinetti, Lundgren, Koltowska-Haggstrom, Petrossians, investigators (21)	792	—	T1, T2	c/s	Yes
Demir, Sulu, Kara, et al (22)	570	1.5-3.0	—	—	—
Amodru, Sahakian, Piazzola, et al (23)	275	1.5	—	—	—
Bier, Hempel, Grimm, Ernemann, Bender, Honegger (24)	159	—	T1, T2	c/s	Yes
Liu, Zhou, Neidert, et al (25)	152	—	T1	c/s	Yes
Potorac, Petrossians, Daly, et al (26)	144	—	T1, T2	—	—
Biagetti, Araujo-Castro, Torre, et al (27)	144	—	T1, T2	c/s	—
Zhang, Chen, Yao, et al (28)	140	3.0	T1, T2	c/s/a	Yes
Park, Kim, Ku, Lee, Kim (29)	132	1.5-3.0	T1, T2	c/s	—
Del Corso, Mesa Junior, Andrade, Fidalski, Boguszewski (30)	117	—	T1, T2	—	—
Colao, Cappabianca, Caron, et al (31)	104	1.0 (min)	T1 (min)	c/s (min)	Yes
Urhan, Hacioglu, Okcesiz, Karaca, Kara, Unluhizarci (32)	102	1.5	T1, T2	c/s/a	Yes
Colao, Pivonello, Auriemma, et al (33)	99	1.0-1.5	T1 GRE	c/s	—
Mercado, Borges, Bouterfa, et al (34)	98	1.0 (min)	T1 (min)	c/s (min)	Yes
Attanasio, Baldelli, Pivonello, et al (35)	92	1.5	T1	c/s	Yes
Abe, Lüdecke (36)	90	—	T1	c/s	—
Ruiz, Gil, Biagetti, et al (37)	81	—	T1, T2	—	Yes
Sathya, Goyal-Honavar, Chacko, et al (38)	81	1.5	T1, T2	c	Yes
Atai, Knudtzon Andersen, Wiedmann, et al (39)	74	1.5-3	T1, T2	c/s	Yes
Godlewska-Nowak, Grochowska, Zieliński, et al (40)	69	1.5	T2	c	—
Cozzi, Montini, Attanasio, et al (41)	67	1.5	—	c/s	—
Gondim, Almeida, de Albuquerque, Gomes, Schops, Ferraz (42)	67	1.5	T1, T2 SE	—	Yes
Caron, Bevan, Petersenn, et al (43)	64	1.5	T1, T2	c/s	Yes
Petersenn, Schopohl, Barkan, et al (44)	60	—	T1	c/s	Yes
Colao, Ferone, Cappabianca, et al (45)	59	1.0	T1	c/s	Yes
Buhk, Jung, Psychogios, et al (18)	45	—	T1 SE/GRE, T2 SE/TSE	c/s	Yes
Shen, Shou, Wang, et al (46)	39	3.0	T1 SE	—	Yes
Maiza, Vezzosi, Matta, et al (47)	36	1.0	T1 SE	c/s	Yes
Colao, Pivonello, Rosato, et al (48)	34	1.5	T1 GRE	c/s	—
Oshino, Saitoh, Kasayama, et al (49)	32	1.5	—	—	Yes
Carlsen, Svartberg, Schreiner, et al (50)	32	1.5	T1	c/s/a	—
Tang, Xie, Guo, et al (51)	32	3	T1, T2, DWI	c	Yes
Suliman, Jenkins, Ross, Powell, Battersby, Cullen (52)	30	—	T1	c/s	Yes
Petersenn, Farrall, De Block, et al (53)	29	—	T1	c/s	Yes
Shimatsu, Teramoto, Hizuka, Kitai, Ramis, Chihara (54)	29	1.5 (min)	T1, T2	c/s	Yes
Pirchio, Auriemma, Vergura, Pivonello, Colao (55)	28	—	—	—	Yes
Attanasio, Lanzi, Losa, et al (56)	27	1.5	T1	c/s	Yes
Bevan, Atkin, Atkinson, et al (57)	27	—	T1	c/s	Yes
Newman, Melmed, George, et al (58)	26	—	—	c/s/a	—
Campbell, Kenning, Andrews, Yadla, Rosen, Evans (59)	26	—	—	—	Yes
Hofstetter, Mannaa, Mubita, et al (60)	24	—	—	—	Yes
Fahlbusch, Keller, Ganslandt, Kreutzer, Nimsky (61)	23	1.5	T1, T2	c/s	No
Amato, Mazziotti, Rotondi, et al (62)	23	0.5	—	—	Yes
Caron, Morange-Ramos, Cogne, Jaquet (63)	22	—	—	c/s/a	
Cozzi, Barausse, Sberna, et al (64)	21	1.5	—	—	—
Cannavò, Squadrito, Curtò, Almoto, Vieni, Trimarchi (65)	20	0.5	—	—	Yes
Luque-Ramirez, Portoles, Varela, et al (66)	19	1.0	T1	—	Yes
Rodriguez-Barcelo, Gutierrez-Cardo, Dominguez-Paez, Medina-Imbroda, Romero-Moreno, Arraez-Sanchez (67)	17	3.0	T1 SE, T2 TSE	c/s/a	Yes
Micko, Wohrer, Wolfsberger, Knosp (68)	14	1.5-3.0	—	—	Yes
Imran, Fleetwood, O’Connell, et al (69)	12	1.5	3D MPRAGE, T1	c/s/a	Yes
Bakker, Verstegen, Manole, et al (70)	10	3.0	T1 TSE/FFE	c/s/a	Yes
Ikeda, Jokura, Yoshimoto (71)	8	1.5	T1	—	—
Chang, Tseng, Chang (72)	7	—	T1	c/s	—
Ceylan, Koc, Anik (73)	7	1.5	—	—	Yes
Sumida, Migita, Tominaga, Iida, Kurisu (74)	6	1.5	T1, T2 SE/FSE	c/s	Yes
Zhang, Yan, Xie, et al (75)	6	3.0	T1, T2	c/s	Yes

List of studies from the systematic review reporting MRI in acromegaly sorted by number of patients.

Abbreviations: a, axial; c, coronal; DWI, diffusion-weighted imaging; FSE, fast SE; GRE, gradient recalled echo; MPRAGE, magnetization-prepared rapid gradient echo; MRI, magnetic resonance imaging; N, Number of patients; s, sagittal; SE, spin echo; T, Tesla; T1, T1-weighted; T2, T2-weighted; TSE, turbo SE.

## Pituitary MRI

MRI, performed with thin sections (maximum 2-3 mm, with no interslice gaps) through the sella, remains the gold standard for imaging of the pituitary gland and surrounding structures (79), as highlighted in all 4 existing consensus guidelines (19, 76-78). With its better soft-tissue contrast, it is superior to CT in distinguishing pituitary adenomas from other tumors of the sellar region and in delineating tumor anatomy, adjacent structures, and the extent of parasellar extension (80, 81).

As somatotroph tumors are most commonly macroadenomas, the degree of suprasellar, infrasellar, and lateral extension is of particular importance for neurosurgical planning, particularly when there is possible cavernous sinus invasion. However, only the ACG guidelines acknowledge the importance of standardized reporting and including information on extension into surrounding structures (19). When considering cavernous sinus invasion, the MRI-based modified Knosp classification provides important information regarding the likelihood of achieving complete adenoma resection and hormonal remission (68, 82, 83) and should be specifically commented on in all formal radiology reports, even when there is no suspected parasellar extension.

Pituitary adenomas typically exhibit slower contrast uptake than the normal gland, resulting in delayed enhancement and washout patterns that can (1) facilitate the identification of otherwise undetectable microadenomas and (2) improve delineation of the boundaries of larger lesions (84). Therefore, most pituitary MRI protocols use gadolinium-based contrast agents (GBCAs). Recent concerns have highlighted potential long-term central nervous system retention of GBCAs, even in patients with normal renal function, which may be significant for patients with pituitary disorders who often require serial longitudinal imaging follow-up (85). The use of macrocyclic GBCAs, with their increased chemical stability, reduces the risk of nephrogenic systemic fibrosis and tissue deposition, although a slightly higher risk of allergic reactions has been reported. Therefore, if non-contrast T1-weighted (T1W) or T2-weighted (T2W) sequences provide sufficient information to inform management, selective omission of gadolinium to minimize exposure may be considered in some contexts (eg, during long-term follow of a stable adenoma tissue remnant) (86). However, setting this concern aside, review of the literature shows that deployment of contrast-enhanced imaging is not uniform, with only 39 (68.4%) of the reviewed MRI studies reporting gadolinium use despite recommendations in consensus guidelines (Table 1) (19, 76-78).

Although MRI is preferred for pituitary and parasellar imaging, when there is intolerance or a contraindication, CT can be considered (76, 77). CT may also provide additional information in certain situations (87). For example, integration of a CT or CT angiogram in the presurgery imaging protocol can facilitate neuro-navigation, delineate sphenoid sinus anatomy and bony erosion by tumor, and aid reconstruction planning if cerebrospinal fluid leak is anticipated (88, 89). Some centers routinely advocate this in all cases (90).

### MRI field strength

Previous MRI studies of somatotroph tumors have described the use of different field strengths, typically ranging from 0.5 Tesla (0.5T) to 3T (Table 1). The ACG guidelines recommend that pituitary tumor centers should have access to an MRI scanner with a field strength of ≥1.5T (19). This provides adequate diagnostic sensitivity in the majority of patients with acromegaly, where macroadenomas predominate and microadenomas are more readily visualized on conventional imaging than their corticotroph counterparts (19, 76). However, with earlier diagnosis, an increased proportion of tumors are likely to be imaged as microadenomas (or even when occult), and in this context, the use of higher field strength MRI may permit more reliable localization (6).

3T MRI may also permit better visualization of the medial wall of the cavernous sinus and the parasellar cranial nerves (91, 92). Infiltration of the cavernous sinus has been reported in approximately one-third of patients with somatotroph adenomas and has important ramifications for surgery and radiotherapy planning and outcomes (93).

Currently, 7T MRI is only available in a limited number of centers. With its superior resolution and improved signal-to-noise and contrast-to-noise ratios, it has the potential to improve localization of small volume de novo, residual, or recurrent disease and has been shown to provide more accurate information on cavernous sinus infiltration with better agreement with intraoperative findings than lower field strength MRI (94). However, to date, it has been used in comparatively few studies of patients with pituitary adenomas, and it remains unclear whether the increased resolution of 7T MRI will lead to an increased rate of false-positive results (95).

### MRI orientation

**Figure 2:**
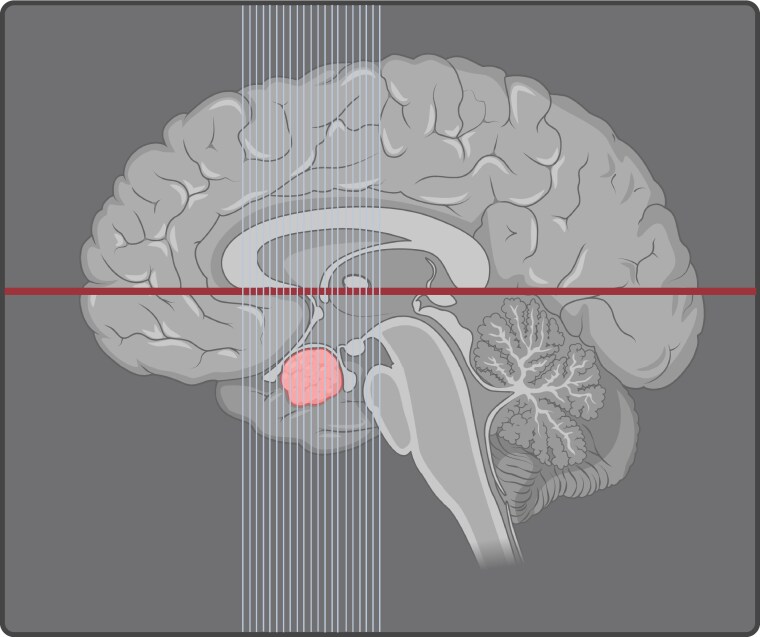
Optimized slice position to acquire sellar and parasellar images. Suggested reference plane for orientation: cutting perpendicular to a horizontal line drawn through the most inferior anterior and posterior aspects of the corpus callosum. Created with BioRender.com and based on information from Bonneville (17).

Routine clinical pituitary imaging includes coronal and sagittal views (Table 1). However, this does not necessarily translate to standardized image acquisition and can present an important challenge for comparison between scans during surveillance follow-up, especially when patient care is transferred between centers. To address this, adoption of imaging protocols and acquisition parameters that are standardized across centers will be required (96). The importance of such an approach is emphasized by the challenges identified during image analyses of the German Pegvisomant Observational Study (97). Bonneville et al have subsequently proposed a practical solution to enable comparisons of images acquired at different timepoints, which allows for reliably reproducible coronal sections, thus reducing misinterpretation due to gantry variations (Fig. 2) (95). Another alternative is to perform volumetric studies, but with the inevitable additional cost and time constraints. In select cases, axial imaging can provide useful additional information for surgical planning and also has particular value during co-registration with molecular imaging (98).

### Standard MRI sequences

**Figure 3:**
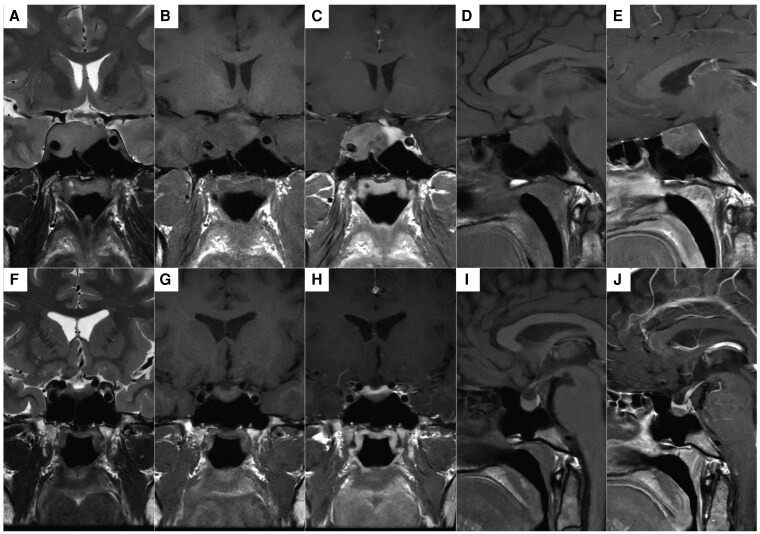
Standard (Tier 1a) MRI in 2 patients with somatotroph adenomas. 1.5T pituitary MRI in 2 patients with somatotroph adenomas. (A-E) Middle-aged man with a macroadenoma demonstrating Knosp 4 extension to the right cavernous sinus: (A) coronal T2W FSE, (B) coronal T1W SE, (C) coronal T1W SE post-gadolinium, (D) sagittal T1W SE, and (E) sagittal T1W SE post-gadolinium. The adenoma is hyperintense to the gray matter of the temporal lobe on T2W sequences (panel A) and shows poor enhancement when compared with the normal gland (left sella), which is best appreciated on the T1W SE post-gadolinium coronal image (panel C). (F-J) Young woman with an inferiorly located mesoadenoma without parasellar extension: (F) coronal T2W FSE, (G) coronal T1W SE, (H) coronal T1W SE post-gadolinium, (I) sagittal T1W SE, and (J) sagittal T1W SE post-gadolinium. The adenoma is hypointense to the gray matter of the temporal lobe on T2W sequences (panel F) and shows poor enhancement when compared with the normal gland (superiorly), which is best appreciated on the T1W SE post-gadolinium images (panels H and J). Abbreviations: FSE, fast SE; MRI, magnetic resonance imaging; SE, spin echo; T, Tesla; T1W, T1-weighted; T2W, T2-weighted.

T1W spin echo (SE) is the standard magnetic resonance (MR) sequence for imaging pituitary adenomas and is typically performed pre- and post-gadolinium injection (Fig. 3). The majority of somatotroph macroadenomas and most microadenomas are readily visualized, with good discrimination between the adenoma, remaining normal gland, and key surrounding structures (95). The American Association of Clinical Endocrinology and ACG guidelines specifically recommend first-line use of this sequence (19, 76).

The Endocrine Society, the Pituitary Society, and the ACG guidelines also highlight the potential added value of T2W fast SE (FSE)/turbo SE (TSE) sequences when visualizing somatotroph tumors (99). T2W imaging provides enhanced delineation of neurovascular structures in the cavernous sinus and can reveal the extent to which a tumor encroaches on, or actually breaches, the medial wall. Axial T2 images can be especially helpful for diagnosis of limited cavernous sinus invasion. It is also increasingly used to predict a likely response to first-generation SRL therapy (eg, octreotide and lanreotide) (76). Studies using a qualitative approach suggest that somatotroph adenomas are most commonly T2 hypointense (especially microadenomas) (17, 26, 99). Moreover, MR signal intensity on T2W sequences has been linked to granulation pattern on pathological analysis of resected tumor specimens, with densely granulated somatotroph adenomas being typically T2 hypointense, consistent with their more favorable response to first-generation SRL therapy (and mediated through somatostatin receptor 2 [SST2] expression on adenoma cells) (26, 100-102) (Fig. 3). This may allow for an assessment of the efficacy of medical treatment before any tumoral volume change can be detected.

However, these findings are not entirely consistent across studies, likely reflecting important methodological differences. For example, in a post hoc analysis of the PRIMARYS study, qualitative assessment of T2 signal intensity led to 59% of tumors being classified as hypointense, whereas quantitative assessment using the signal-ratio method (signal intensity ratio of the adenoma vs the adjacent gray matter) identified only 36% as hypointense (100). Another quantitative method comparing the signal intensity ratio of the adenoma with adjacent gray and white matter (3-tissue method) classified only 20% as hypointense (100).

Accepting these challenges, T2 signal intensity remains a useful tool to anticipate first-generation SRL therapy response in many patients (99, 101). However, with multi-receptor–targeted pasireotide, the link between T2 signal intensity and treatment response (biochemical control vs tumor shrinkage) is less clear. In 69% of patients treated with pasireotide, T2 isointensity or hyperintensity on qualitative assessment was shown (15). Notably, a higher baseline T2 MRI tumor intensity predicted a higher rate of biochemical control, but not adenoma shrinkage (15). However, in the study of Ruiz et al, biochemical control and significant tumor shrinkage in response to pasireotide were not associated with initial T2 intensity (37).

In the future, the use of T2W images in predictive diagnostics in acromegaly will likely be augmented via the use of a machine-learning approach with quantitative texture analysis. In a retrospective study by Kocak et al, this approach correctly classified response to SRL therapy in over 80% of cases, surpassing conventional qualitative and quantitative relative T2 signal intensity, and showed superiority to immunohistochemical evaluation. However, the latter was based only on cytokeratin staining with classification as sparsely and non-sparsely granulated adenomas and did not include SST staining (103). Using a different approach, Park et al identified radiomic features that enabled prediction of the granulation pattern of somatotroph adenomas and thereby likely first-generation SRL responsiveness (104). However, for the moment, the visual method appears to offer a simple and valid approach to initial T2 signal intensity assessment, although further standardization of reading and reporting of scans would likely increase the accuracy of even this simple methodology (40, 105).

### Additional MRI sequences

Advanced volumetric sequences such as 3-dimensional (3D) gradient recalled echo (GRE) and 3D FSE can provide improved soft-tissue contrast and aid discrimination between residual adenoma, postoperative tissue remodeling, and normal gland (106, 107). These sequences use isotropic voxels (ie, length, width, and height identical), which allow for effective volumetrics and segmentation, making them particularly useful for intraoperative neuro-navigation. In this context, spoiled T1W GRE techniques (eg, magnetization-prepared rapid gradient echo [MPRAGE] and fast spoiled gradient echo [FSPGR]) offer additional benefits, with volumetric interpolated breath-hold examination (VIBE) showing the highest consensus with intraoperative findings of tumor invasiveness in a prospective case control study (108). Volumetric sequences may enhance the detection of occult somatotroph microadenomas (analogous to microcorticotropinomas in Cushing disease) and should also be considered at an early stage in patients with macroadenomas, especially when surgery/radiosurgery is being planned. Dynamic contrast-enhanced MRI, while not recommended as a routine first-line sequence, may be considered selectively in occult or equivocal lesions.

High contrast resolution balanced steady-state GRE sequences (eg, constructive interference in steady state [CISS] or fast imaging employing steady-state acquisition [FIESTA]) provide enhanced visualization of the cavernous sinus structures and cranial nerves (109). Assessment of Knosp grades using CISS, when compared with standard imaging, has been reported to predict cavernous sinus invasion more accurately (110, 111).

Contrast-enhanced FIESTA can improve delineation of the anterior visual pathways (112), and optic nerve kinking as assessed by FIESTA might be a predictive factor for irreversible damage to the visual system (113). Edema and microstructural damage within the visual pathways can be detected by diffusion tensor imaging (DTI), which provides additional predictive value when combined with optical coherence tomography (OCT) (114).

MR elastography (MRE) combines structural imaging with low-frequency vibrations to create visual maps, which can provide insights into adenoma consistency ahead of surgery. This allows for better prediction of operation time and success probability during surgical planning (98, 115, 116). Although diffusion-weighted imaging (DWI) has been proposed as an alternative method for assessing tumor consistency, lack of reproducibility between studies has raised doubts about its reliability (117). For example, while the apparent diffusion coefficient in DWI has been reported by some workers to correlate with pituitary adenoma consistency (118, 119), other groups have not replicated these findings (120-122). DWI-based virtual MRE could provide a solution to the challenges of DWI, especially in heterogeneous lesions, and in 1 study, it accurately predicted tumor consistency in a series of 10 patients (123).

Various intracranial internal carotid artery changes have been reported in acromegaly, including aneurysms, a narrowed distance between the internal carotid arteries, carotid artery protrusion, and a dehiscent parasellar carotid bony canal. In these contexts, techniques such as MR angiography (MRA) may provide useful information regarding arterial anatomy (124).

Finally, 1 group has explored the possibility of using MR spectroscopy (MRS) to predict response to SRL therapy in somatotroph adenomas, although it remains unclear whether this is of additional value when compared to T2W imaging alone (125).

### Specific imaging considerations at different time points

#### Diagnosis

Consensus guidelines recommend performing MRI with gadolinium enhancement once the biochemical diagnosis of acromegaly has been established to visualize tumor size, appearance, and parasellar extension (19, 76, 77). Neuroimaging should not be used in lieu of biochemical diagnosis due to the potential for confounding by pituitary incidentalomas (126).

#### Intraoperative

Intraoperative MRI (iMRI), which is available in some surgical centers, may aid differentiation between residual tumor and blood products during surgery, and thereby facilitate more complete tumor removal (61). Gross total resection has been reported in an additional 18% to 30% of patients (127, 128), with iMRI providing additional benefit in secretory tumors (129), particularly when combined with endoscopy and neuronavigation (130). However, it is less clear if tumor size is a relevant factor for predicting the utility of iMRI. While current evidence points to a particular benefit for adenomas with Knosp grade < 3 (129, 131), there are also data to support a role for the use of iMRI in larger tumors (132).

Importantly, use of iMRI increases the duration of surgery, and outcomes are significantly influenced by patient selection and neurosurgical expertise. Currently, its use remains restricted to individual cases in specialized centers (133).

Intraoperative ultrasound has been suggested as a safe and effective alternative to identify hidden adenomas, improving tumor removal rates and minimizing the risk of damage to nearby vascular structures (134). Larger, multicenter studies conducted in Pituitary Tumor Centers of Excellence (PTCOEs) will be required to confirm its potential added benefit, particularly for surgeons who already achieve high success and low complication rates in microadenoma resections.

More recently, small pilot studies have explored innovative techniques for real-time intraoperative tumor visualization. In the DEPARTURE trial, Schmidt et al investigated quantitative fluorescence molecular endoscopy using bevacizumab-800CW, a fluorescent tracer combining a humanized anti-vascular endothelial growth factor-A monoclonal antibody with a near-infrared dye (135). This non-randomized, open-label, single-center feasibility study, which included 19 patients with cavernous sinus invasion (including 2 with somatotroph tumors), demonstrated that quantitative fluorescence molecular endoscopy was a safe and effective method to distinguish tumor tissue from normal structures. In a separate first-in-human pilot study, Waterhouse et al used intraoperative multispectral imaging with an endoscope-mounted multispectral camera to differentiate normal pituitary tissue from adenomas (136). Further refinement and optimization of imaging parameters will be necessary before this technology can be assessed for its full clinical potential.

#### Follow-up

In the recent update describing criteria for diagnosis and remission, the ACG sets out several key considerations for follow-up imaging (19). For most patients, MRI using standard clinical sequences is recommended 3 to 6 months after surgery to establish a new baseline for future reference (76-78). Some experts specifically advocate imaging closer to 6 months to minimize the risk of confounding by postoperative changes. Repeat imaging is also advised whenever a change in therapeutic modality is considered (78). For routine surveillance, gadolinium administration should not be considered mandatory, and indeed, it has been proposed that it should only be used when uncertainty persists on non-contrast images (76).

##### When is less frequent or no routine MRI surveillance indicated?

Following the establishment of a posttreatment new baseline MRI, the frequency of repeat imaging should be guided by the degree of biochemical control, tumor behavior, and overall clinical context. For example:

- Patients in sustained biochemical and clinical remission after surgery may not require routine MRI surveillance, especially when the original tumor was a microadenoma (137).
- Less frequent or no further imaging may be reasonable in patients who are older or frail with significant comorbidities, particularly when disease control is readily achieved and maintained on medical therapy.
- However, late recurrences can occur even after long disease-free intervals (≥10 years) (138), and therefore, continued but less frequent imaging should be considered in selected cases (87).

##### When is more frequent MRI surveillance indicated?

More frequent MRI follow-up should be considered for patients with a higher risk of tumor regrowth or when there is uncertainty about disease control. For example:

- Following surgery, if biochemical control is incomplete, or there are unanticipated fluctuations in disease activity.
- If there is evidence of persistent disease activity despite optimized medical therapy, an MRI should be obtained to exclude concomitant adenoma regrowth (6).
- Patients with aggressive adenomas or underlying genetic syndromes should also undergo closer and individualized imaging surveillance (19).

In patients receiving growth hormone (GH) receptor antagonist therapy, surveillance MRI is recommended at 3 to 6 months post-initiation. Earlier guidelines advised continued annual imaging due to concerns about tumor re-expansion following SRL withdrawal and potential growth, while taking GH receptor antagonist therapy. However, findings from the large ACROSTUDY dataset show a low incidence (∼3%) of tumor growth that was comparable to that seen in untreated acromegaly (20, 97, 139). Consequently, current ACG guidelines advocate an individualized approach, taking into account country-specific labelling requirements, adenoma characteristics, and biochemical profile (19).

Importantly, there is a need for consistency in imaging protocols and acquisition parameters across follow-up studies to permit greater confidence in confirming or refuting concerns regarding adenoma growth (97, 139).

### Special circumstances: pregnancy and genetic disease

Consensus guidelines recommend avoiding routine MRI surveillance during pregnancy, unless there is evidence of new or worsening visual field compromise (76). If MRI is performed (eg, when there are concerns regarding tumor expansion with visual loss), gadolinium enhancement is not advised (76).

In the rare patients with acromegaly associated with germline mutations in genes such as *AIP* or *MEN1*, MRI findings may indicate a more aggressive pituitary adenoma phenotype (140). Individuals harboring *AIP* mutations typically present at a younger age and are more likely to have large macroadenomas at diagnosis, often demonstrating invasive characteristics such as cavernous sinus involvement or suprasellar extension (141). Although the MRI features of MEN1-related somatotroph adenomas are less well defined, these lesions likewise tend to be larger, multifocal, and more resistant to conventional therapy compared with sporadic cases. Accordingly, MRI in the setting of a known genetic predisposition to acromegaly serves to delineate tumor burden and anatomic invasiveness prior to surgery, as well as inform closer longitudinal surveillance of mutation carriers, even when baseline imaging is unremarkable.

## Pituitary molecular imaging

Molecular (functional) imaging has a long history in the diagnostic evaluation of somatotroph adenomas, with somatostatin receptor scintigraphy (SSTS) using indium-111 octreotide approved by the US Food and Drug Administration in 1994 (142). However, in the context of somatotroph adenomas, the utility of SSTS is limited by the high physiological uptake of radioligand by normal underlying pituitary tissue, which can obscure lesion detection, coupled with the limited spatial resolution of scintigraphy and single-photon emission CT (SPECT) (143, 144). Moreover, clinical studies have not consistently demonstrated a predictive role for SSTS in assessing response to SRL therapy (145-147). Although some reports have suggested that SSTS may offer incremental diagnostic value in selected cases of somatotroph adenomas (148), its role appears most relevant in rare instances of ectopic acromegaly (149). In such cases, SSTS may be considered alongside thoracic and abdominal imaging when biochemical evidence of acromegaly is accompanied by pituitary hyperplasia or an empty sella (77). Importantly, however, the most recent ACG guidelines underscore that a comprehensive review of all sella imaging by an expert neuroradiologist within a PTCOE should precede any search for an ectopic hormonal source (19).

The increasing availability of PET, with its significantly higher spatial resolution and improved sensitivity for lesion detection, has led to a resurgence of interest in the use of molecular imaging to guide treatment decisions in acromegaly, especially in the context of residual or recurrent pituitary disease following primary intervention.

### ^68^Gallium-somatostatin receptor (^68^Ga-SST) PET

In principle, ^68^Ga-SST PET (using ^68^Ga-DOTATATE, ^68^Ga-DOTATOC, or ^68^Ga-DOTANOC) offers receptor-targeted functional information to complement high-resolution MRI. However, as with earlier approaches using scintigraphy and SPECT, physiological SST2 binding in normal anterior pituitary tissue and treatment-related receptor occupancy (eg, during SRL therapy) can blunt lesion-to-background contrast, especially for microadenomas (150). In a small prospective study, Daniel et al evaluated ^68^Ga-DOTATATE uptake in the pituitary region of patients with acromegaly and its relationship to biochemical response following SRL therapy (151). They found no significant difference in pituitary maximum standardized uptake values between treatment responders and nonresponders. However, an inverse correlation between postoperative GH levels and pituitary ^68^Ga-DOTATATE uptake suggested that tracer binding may reflect SST2 expression. Overall, ^68^Ga-DOTATATE uptake did not reliably predict therapeutic response but may have limited value as a biomarker of receptor density (151). Contemporary expert guidance echoes these nuances: while SST-targeted tracers may help in selected “problem-solving” scenarios—such as coregistration with MRI to delineate residual disease for radiosurgical planning (152)—current evidence is insufficient to recommend routine ^68^Ga-SST PET over high-quality MRI for primary sellar localization in patients with acromegaly (153).

### ^11^C-methionine PET (MET-PET) and ^18^F-fluoroethyltyrosine PET (FET-PET)

^11^C-Methionine-PET (MET-PET; performed either as hybrid MET-PET/MR or using MET-PET/CT co-registered with volumetric MRI [MET-PET/MR^CR^]) has emerged as the most effective molecular imaging modality to evaluate pituitary adenomas in acromegaly. Several groups have demonstrated the diagnostic accuracy of MET-PET in localizing residual or recurrent somatotroph adenomas (14, 67, 154). In the study by Rodríguez-Barceló et al (67), MET-PET achieved a sensitivity of 86% and a specificity of 100% for identifying residual pituitary adenoma. In comparison, ^18^F-fluorodeoxyglucose PET (^18^F-FDG-PET) demonstrated a considerably lower sensitivity of 28% despite maintaining equivalent specificity (67).

The mechanism underlying this diagnostic advantage lies in the active uptake of methionine via the L-type amino acid transporter 1, which is expressed in metabolically active adenoma tissue. Somatotroph adenomas typically demonstrate strong methionine avidity, akin to that observed in prolactin-secreting tumors (155, 156). However, radioligand uptake may be significantly attenuated by concomitant medical treatment. For example, both SRL and dopamine agonist therapy can suppress somatotroph tumoral activity and thereby diminish radioligand uptake. Accordingly, optimization of molecular imaging is dependent on performing a scan when disease activity has clearly been demonstrated. In those receiving medical therapy capable of directly suppressing tumor function, a period of drug washout is advised (this may require several months for depot SRL therapy and ≥1 month for longer-acting dopamine agonist therapy). In contrast, agents with no direct pituitary-targeted effects, such as GH receptor antagonist therapy, can be safely continued or substituted to prevent symptom recurrence.

Consistent with this, a single-center prospective series reported that MET-PET/MR^CR^ successfully localized residual somatotroph adenoma in 25 of 26 patients with persistent hormonal hypersecretion following prior treatment. PET-guided transsphenoidal surgery was performed in 14 of these individuals, leading to substantial improvements in hormonal control; notably, the single false-negative scan occurred in a patient who remained on SRL therapy before imaging (14, 157). MET-PET was also useful in delineating the true lateral extent of residual adenoma, thereby assisting in surgical planning and reducing the risk of incomplete resection (14).

Further evidence derived from an expanded cohort at the same institution has confirmed these findings. Among patients with inconclusive or negative MRI results, MET-PET identified 52 lesions, enabling (repeat) transsphenoidal surgery in 33 cases. Of these, 24 patients achieved complete biochemical remission, while only 3 experienced new, single pituitary hormone deficits, highlighting the procedure's diagnostic and therapeutic impact (158).

However, despite its clinical promise, the use of MET-PET remains limited by logistical constraints, as the tracer's short half-life (∼20 minutes) necessitates an on-site cyclotron. To overcome this barrier, alternative radioligands targeting the same L-type amino acid transporter 1 have been proposed, including FET-PET, which benefits from a longer half-life (∼110 minutes) and increased availability (the radioligand can be synthesized centrally and distributed to multiple sites). Preliminary case reports and small case series suggest similar tumoral uptake patterns, although the evidence base remains small and larger comparative studies are required to validate its diagnostic performance in acromegaly (70, 159).

In summary, MET-PET represents a highly sensitive and specific adjunct to MRI for the localization of somatotroph adenomas, with a growing body of evidence supporting its central role in guiding the decision of whether to offer repeat surgery (or radiosurgery) in patients with persistent or recurrent disease following primary treatment. Development of longer-lived amino acid tracers such as FET may increase accessibility to molecular imaging and broaden its clinical application in centers without cyclotron facilities.

## Proposal of a tiered approach to pituitary imaging in acromegaly

**Figure 4:**
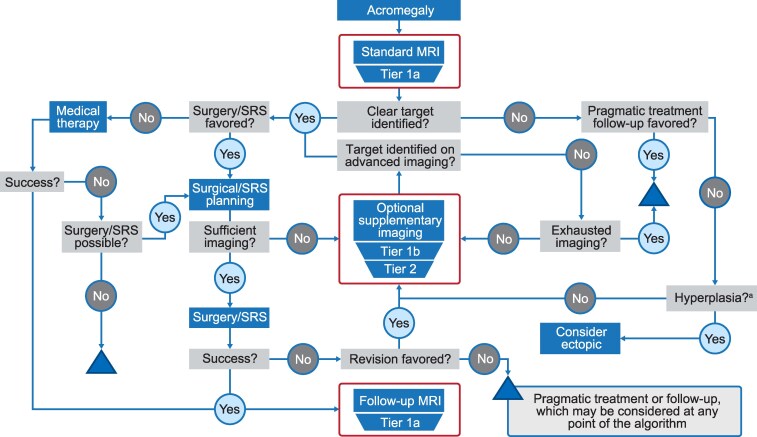
Proposed tiered imaging algorithm for acromegaly. **Tier 1a:** minimum 1.5T, maximum 2- to 3-mm slice thickness (with no interslice gap) T1W SE in coronal and sagittal planes with and without gadolinium enhancement, and T2W FSE/TSE MRI. **Tier 1b:** Higher field strength (3T) or 3D T1W spoiled gradient recalled echo. **Tier 2:** Advanced MRI sequences (eg, fast imaging employing steady-state acquisition [FIESTA]) or other imaging modalities (eg, molecular imaging with MET-PET)—typically limited to specialist centers (PTCOEs). Applications and options for Tier 1b and Tier 2 imaging are further illustrated in Fig. 5. **Key:**  ^a^Pituitary demonstrates enlarged, hyperplastic appearance; triangle symbol denotes decision for pragmatic treatment or follow-up, which may be considered at any point in the algorithm. Abbreviations: 3D, 3-dimensional; FIESTA, fast imaging employing steady-state acquisition; FSE/TSE, fast/turbo SE; MET, ^11^C-methionine; MRI, magnetic resonance imaging; PET, positron emission tomography; PTCOEs, Pituitary Tumor Centers of Excellence; SE, spin echo; SRS, stereotactic radiosurgery; T, Tesla; T1W, T1-weighted; T2W, T2-weighted. Created with BioRender.com. A digital version of this algorithm can be found at www.acroimaging.com.

While most macroadenomas can be readily visualized using modern 1.5T MRI scanners, the adoption of common imaging standards would be expected to increase diagnostic efficacy and help ensure that radiologic studies of this rare condition can be more readily compared between centers (18). If the use of a standard MRI protocol does not show a clear target for surgery or radiotherapy (including stereotactic radiosurgery), further imaging options can be considered. Based on the published literature and our extensive clinical experience, we now propose a tiered approach to acromegaly imaging, sequentially employing more advanced options as needed. A diagnostic algorithm incorporating advanced imaging is shown in Fig. 4. For ease of use, we also provide a digital version on AcroImaging.com.

For core diagnostic imaging (Tier 1a), high-resolution T1W SE MRI in both sagittal and coronal planes—acquired before and after gadolinium administration—is recommended. Imaging should employ thin contiguous slices (2-3 mm, no interslice gap) and a maximum 512 matrix to optimize spatial resolution and lesion conspicuity. Complementary T2 FSE/TSE sequences in the coronal plane (with optional sagittal or axial acquisition) provide valuable additional information, particularly in characterizing cystic, hemorrhagic, or necrotic tumor components. Moreover, T2 signal characteristics may carry prognostic significance, as they have been linked with SRL responsiveness in somatotroph adenomas (Fig. 3).

**Figure 5:**
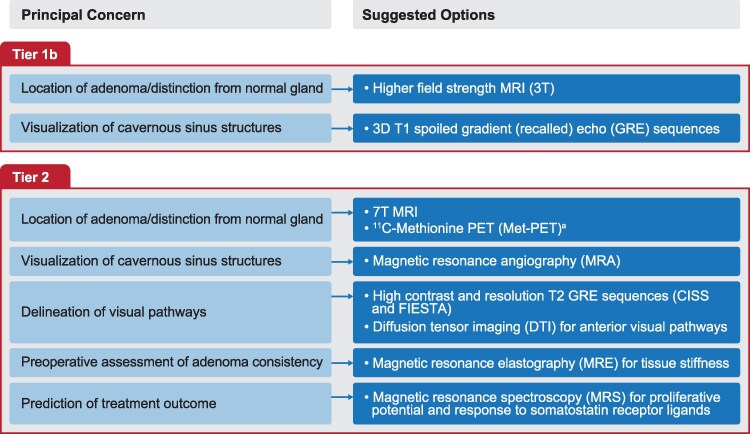
Examples of advanced imaging modalities with potential utility in somatotroph adenoma management. ^a^Alternative radioligands targeting the L-type amino acid transporter 1 (eg, ^18^F-FET) may also be considered for molecular imaging. Abbreviations: 3D, 3-dimensional; ^18^F-FET, ^18^F-fluoroethyltyrosine; CISS, constructive interference in steady state; DTI, diffusion tensor imaging; FIESTA, fast imaging employing steady-state acquisition; GRE, gradient recalled echo; MET, ^11^C-methionine; MET-PET/MR^CR^, MET-PET/computed tomography co-registered with volumetric MRI; MRA, magnetic resonance angiography; MRE, magnetic resonance elastography; MRI, magnetic resonance imaging; MRS, magnetic resonance spectroscopy; PET, positron emission tomography; T, Tesla.

In patients with occult (de novo or residual) pituitary tumor or for preoperative surgical planning, the use of higher field strength MRI (3T if previous imaging performed at 1.5T), dynamic contrast-enhanced sequences and, where appropriate, 3D T1W spoiled gradient-echo imaging to assess cavernous sinus invasion should be considered. These techniques, classified as Tier 1b imaging, are likely available in most tertiary centers and offer improved anatomical definition and contrast resolution compared with standard protocols. In contrast, more advanced imaging modalities (Tier 2)—such as hybrid PET/MR and other specialized functional imaging approaches—are likely to be limited to PTCOEs (160), ensuring appropriate expertise to guide management in these more complex cases. A flowchart summarizing the advanced imaging strategies included within Tiers 1b and 2 is presented in Fig. 5.

When a pituitary lesion is identified on initial MRI and the multidisciplinary team, in agreement with the patient, elects for surgical management, further imaging to refine surgical planning may be warranted. Detailed assessment of the adenoma relationship to critical structures—including the cavernous sinus, optic chiasm, and adjacent cranial nerves—can be enhanced using advanced imaging modalities. This includes MRA for visualization of cavernous sinus vasculature, high-resolution T2W GRE sequences, and DTI to delineate the visual pathways. The use of isotropic volumetric sequences facilitates accurate neuro-navigation, while additional sequences, such as MPRAGE or FSPGR, may assist in improved vascular and soft-tissue definition. The decision to deploy iMRI should be based on local expertise, infrastructure, and workflow feasibility.

For occult primary or equivocal residual disease, we recommend the use of higher field strength MRI—either 3T (Tier 1b) or 7T (Tier 2)—alongside advanced structural sequences such as 3D GRE or 3D FSE (Tier 1b) or, where available, molecular imaging modalities such as MET-PET (Tier 2). These techniques can improve lesion detection and help distinguish residual tumor from normal pituitary tissue or posttreatment tissue remodeling. Such approaches are also valuable prior to stereotactic radiosurgery, ensuring maximal targeting accuracy and treatment precision (152).

If the pituitary gland appears diffusely enlarged or hyperplastic, ectopic GH-releasing hormone secretion should be considered as a possible rare cause. Although the finding of an empty sella on initial MRI in newly diagnosed acromegaly has been proposed as a potential indicator of ectopic disease, in most cases of suspected empty sella, advanced sellar imaging is recommended to exclude the presence of a small, otherwise occult pituitary microadenoma located in the tissue rim, before embarking on a search for a rare ectopic tumoral origin (24, 25, 161, 162).

**Figure 6:**
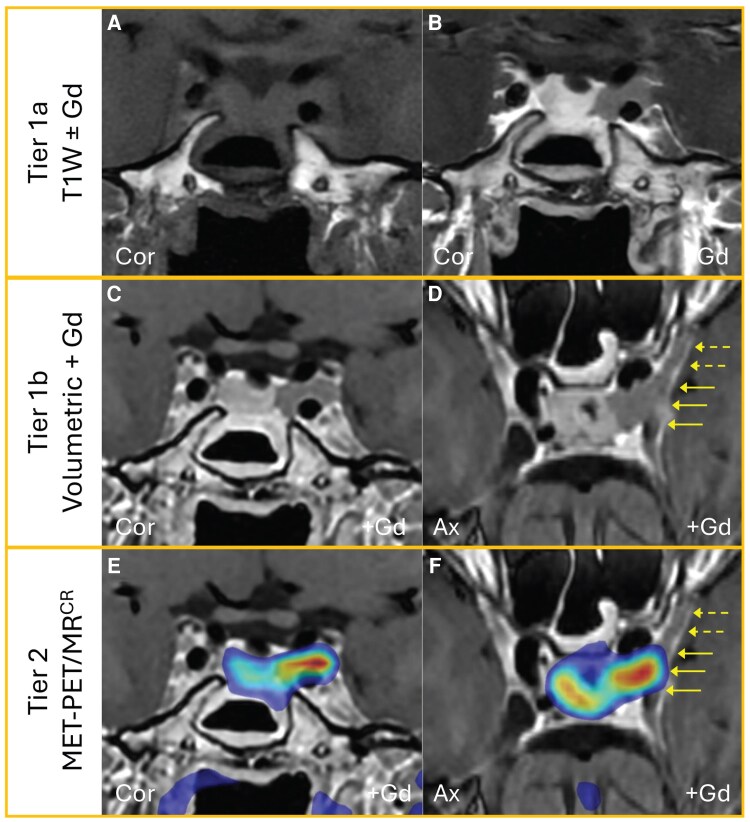
Illustration of the application of a tiered approach to imaging in acromegaly. A middle-aged woman with recurrence of her acromegaly wished to explore the potential for further transsphenoidal surgery in a PTCOE. (A-B) Standard T1W SE MRI demonstrated tumor within the left sella extending into the left cavernous sinus (Knosp 3A) (Tier 1a; T1W SE coronal pre- and post-gadolinium). (C-D) 3D FSE volumetric sequences suggested extension to abut the lateral wall of the cavernous sinus (solid arrows) but also raised the possibility of anterior extension (dashed arrows) (Tier 1b; volumetric FSE coronal and axial planes post-gadolinium). (E-F) MET-PET revealed less Knosp 3A extension than initially suspected and excluded anterior extension of the tumor (Tier 2; MET-PET/MR^CR^ in coronal and axial planes). Abbreviations: 3D, 3-dimensional; Ax, axial; Cor, coronal; FSE, fast SE; Gd, gadolinium; MET, ^11^C-methionine; MET-PET/MR^CR^, MET-PET/computed tomography co-registered with volumetric MRI; MRI, magnetic resonance imaging; PET, positron emission tomography; PTCOE, Pituitary Tumor Center of Excellence; SE, spin echo; T, Tesla; T1W, T1-weighted.

An illustrative example of a patient who underwent Tier 1a, Tier 1b, and Tier 2 imaging is shown in Fig. 6. Ultimately, decisions regarding the application of advanced imaging modalities should be individualized, considering local expertise, resource availability, and patient preference, while maintaining a pragmatic, multidisciplinary approach in cases where diagnostic uncertainty persists.

## Future outlook

As 7T MRI and molecular imaging become more widely available, advances in post-acquisition image reconstruction and analysis will be central to harnessing the full potential of these imaging modalities.

Increasingly, machine learning approaches are being applied to imaging diagnostics, with deep learning techniques for image reconstruction implemented in more modern scanners. Such approaches have the potential to significantly increase the signal-to-noise ratio and enable even more granular data acquisition (eg, through thin [≤1 mm] slicing) (163). This, in turn, should lead to the detection of even smaller microadenomas and aid better assessment of cavernous sinus invasion. Machine learning algorithms for segmentation of the sellar region have also been reported (164). By leveraging these algorithms, the pituitary multidisciplinary team can obtain more accurate, efficient, and cost-effective detection of sellar lesions, thereby aiding surgical planning and monitoring of response to therapy.

In addition to, and aided by, machine learning algorithms, radiomics has emerged as a promising approach in imaging diagnostics. By analyzing and evaluating imaging parameters that are beyond the appreciation of the human eye, radiomics can provide valuable insights into therapy outcomes and tumor characteristics for pituitary adenomas (165, 166), resulting in a more personalized and precise approach to acromegaly treatment.

## Conclusions

As therapeutic options for acromegaly continue to expand, there is growing emphasis on delivering individualized, cost-effective treatment strategies that optimize biochemical control while minimizing adverse effects and health care expenditure. Within this evolving landscape, high-quality imaging remains integral to accurate diagnosis, treatment selection, and longitudinal monitoring. Despite its central role, standardization and optimization of sellar imaging protocols have received comparatively little attention, although suboptimal imaging can directly compromise clinical decision-making and patient outcomes. Furthermore, inter-center and intra-center variability in scanning techniques and image acquisition parameters continue to limit the comparability of studies, impeding both consistency in clinical decision-making and research collaboration.

We therefore propose a stepwise, standardized framework for sellar and parasellar imaging in acromegaly, designed to be readily implementable across pituitary centers. Adoption of such an approach should enhance the quality and reproducibility of diagnostic imaging and also facilitate collaborative multicenter research, thereby advancing evidence-based management and improving outcomes for patients living with acromegaly.

## Data Availability

Data sharing is not applicable to this article as no data sets were generated or analyzed during the present study.

## Abbreviations

3D: 3-dimensional
ACG: Acromegaly Consensus Group
CISS: constructive interference in steady state
CT: computed tomography
DTI: diffusion tensor imaging
DWI: diffusion-weighted imaging
^18^F-FDG: ^18^F-fluorodeoxyglucose
FET: ^18^F-fluoroethyltyrosine
FIESTA: fast imaging employing steady-state acquisition
FSE: fast spin echo
FSPGR: fast spoiled gradient echo
^68^Ga-SST: ^68^Gallium-somatostatin receptor
GBCA: gadolinium-based contrast agent
GH: growth hormone
GRE: gradient recalled echo
iMRI: intraoperative magnetic resonance imaging
MET: ^11^C-methionine
MET-PET/MR^CR^: ^11^C-methionine-positron emission tomography/computed tomography co-registered with volumetric magnetic resonance imaging
MPRAGE: magnetization-prepared rapid gradient echo
MR: magnetic resonance
MRA: magnetic resonance angiography
MRE: magnetic resonance elastography
MRI: magnetic resonance imaging
MRS: magnetic resonance spectroscopy
OCT: optical coherence tomography
PET: positron emission tomography
PTCOE: Pituitary Tumor Center of Excellence
SE: spin echo
SPECT: single-photon emission computed tomography
SRL: somatostatin receptor ligand
SRS: stereotactic radiosurgery
SST: somatostatin receptor
SSTS: somatostatin receptor scintigraphy
T: Tesla
T1: T1-weighted
T2: T2-weighted
TSE: turbo spin echo
VIBE: volumetric interpolated breath-hold examination
